# Experiences and perceptions of referrals to a community-based physical activity program for cancer survivors: a qualitative exploration

**DOI:** 10.1186/s12913-021-06365-9

**Published:** 2021-04-17

**Authors:** Jamie M. Faro, Kristin M. Mattocks, Dalton Mourao, Catherine S. Nagawa, Stephenie C. Lemon, Bo Wang, Sarah L. Cutrona, Rajani S. Sadasivam

**Affiliations:** 1 Department of Population and Quantitative Health Sciences, University of Massachusetts Medical School, Worcester, 368 Plantation Street, Worcester, MA 0160 USA; 2 VA Central Western Massachusetts Healthcare System, Leeds, MA USA; 3 Center for Healthcare Organization and Implementation Research, Edith Nourse Rogers Memorial Veterans Hospital, Bedford, MA USA

**Keywords:** Cancer, Physical activity, Referrals, Community, Providers

## Abstract

**Background:**

Physical activity rates in cancer survivors continue to be low despite the known benefits and availability of evidence-based programs. LIVESTRONG at the Y is a national community-based physical activity program offered cost-free to cancer survivors, though is underutilized. We explored perceptions and experiences of staff and participating survivors to better understand program awareness, referrals and participation.

**Methods:**

LIVESTRONG at the Y program staff [directors (*n* = 16), instructors (*n* = 4)] and survivors (*n* = 8) from 8 United States YMCAs took part in 30-min semi-structured phone interviews between March–May 2019. Interviews were digitally recorded, transcribed, and evaluated using a thematic analysis approach.

**Results:**

Program staff themes included: 1) Program awareness should be further developed for both the general public and medical providers; 2) Strong relationships with medical providers increased program referrals; 3) Electronic referral systems between providers and LIVESTRONG would help to streamline the referral process; and 4) Bi-directional communication between program staff and medical providers is key to providing patient progress updates. Survivor themes included: 1) Survivors trust their medical team and the information they provide about physical activity; 2) Providers need to incorporate an action plan and referrals for survivors to be active once treatments are completed; and 3) Personal experiences of those who participated in LIVESTRONG resonate with survivors and increase participation.

**Conclusions:**

LIVESTRONG staff reported the need for an integrated electronic referral system and bi-directional communication with providers about participant progress. Survivors want physical activity education, electronic referrals and follow-up from their healthcare team, coupled with peer support from other survivors. Cancer care provider knowledge and electronic referrals during and after treatment may expedite and increase participation in this community-based program.

## Background

Among cancer survivors, regular physical activity may prevent cancer recurrence and lead to improvements in quality of life, fatigue, fitness, body composition, mood, self-esteem and physical function [1, 2]. The American Cancer Society and the American College of Sports Medicine recommend that cancer survivors aim to achieve 150 min of moderate physical activity per week [3, 4]. Despite these recommendations, less than 30% of survivors are meeting physical activity guidelines [5]. Many survivors lack survivorship care plans with clear instructions for physical activity. Others experience geographic limitations (lack of access to comprehensive cancer exercise centers, lack of nearby in-person programs, unsafe neighborhoods) or financial constraints limiting access [6]. The Institute of Medicine recommends cancer survivorship care plans be provided to all patients, with information on lifestyle recommendations and physical activity prescriptions plus referrals [7]. To be effective, these referrals should ideally connect survivors to affordable, accessible resources.

LIVESTRONG at the Y is a national, community-based program that addresses financial and access barriers to survivor physical activity. Any person with a past cancer diagnosis, over the age of 18 years and medically cleared to perform physical activity is eligible to join. It is a cost-free evidence-based physical activity program shown to be safe and effective at increasing activity levels (71% exercising at ≥ 150 min/week vs 26% of controls; *P* < .05), quality of life and fitness for survivors [8]. The program meets in a small group format twice per week for 12 weeks and is led by specially trained LIVESTRONG at the Y instructors [9]. Instructors create tailored programs addressing the medical, social and physical activity needs of each survivor. This includes components of fitness (aerobic, strength, flexibility and balance) in addition to social support (positive support, active listening, shared storytelling) [9]. As of 2018, an estimated 62,000 people had completed the program at over 240 locations in 42 states [10]. Still, those who have completed LIVESTRONG at the Y represent a small fraction (0.004%) of the more than 17 million survivors nationwide [10]. The most frequently reported method of hearing about the program from past participants was through referral from a doctor or other healthcare professional (27%) followed by word-of-mouth from a friend or family member (22%), followed by other reported sources [9]. Prior studies have reported that patients would like to hear about physical activity from an exercise specialist at the cancer center, or from their oncologist [6]. However, cancer care providers have reported lack of awareness of community-based programs within large areas served by hospitals, thereby reducing referrals to the program [11].

The aim of this study was to explore perceptions of and experiences with the referral process for the community-based physical activity program, LIVESTRONG at the Y. We studied both currently employed program staff and survivors who had previously completed the program. Gaining a better understanding of these experiences is essential to improve strategies and processes designed to refer survivors to community-based programs.

## Methods

### Setting and sample

To ensure a variety of perspectives were captured, we conducted a qualitative study of LIVESTRONG at the Y Program Directors, instructors, and recent program graduates using 30-min semi-structured interviews between March and May 2019. Specific inclusion criteria included: 1) current LIVESTRONG at the Y Program Director or in a position of program leadership/supervisory role, 2) current LIVESTRONG at the Y Instructor, and 3) recent (within 3-years) graduate of LIVESTRONG at the Y. Participants were recruited in collaboration with YMCA-USA leadership, a group responsible for overseeing implementation of all the YMCA’s chronic disease prevention programs. YMCA-USA leaders sent all program directors an email of support regarding this study with contact information for our research team. The research team was granted access to the master list of program directors contact information. In prior work, we identified sites located in both low and high household income areas based on a median of $47,300 [10]. Using these data, we purposefully sampled directors from branches in both low household (*n* = 7) and in high household income areas (*n* = 9) to ensure a representative sample by location. Emails were sent to 25 directors, across 15 US states. Of the 25 emails sent, 2 were undeliverable, thus out of 23 delivered emails, 16 program directors (69.6%) completed the study. Due to the location of the research team’s site, a greater sample of directors in Massachusetts (*n* = 7) were chosen based on prior relationships and potential sites for future work. Once a program director completed an interview, they were provided with a recruitment email from the study team to pass on to program instructors and/or cancer survivors who took part in the program. Interested instructors and survivors contacted the study team via telephone or email to perform a phone screening and subsequent interview if eligible. The lead researcher and interviewer (JF) were trained in conducting qualitative interviews. This study was approved by the Institutional Review Board at the University of Massachusetts Medical School.

### Data collection and analyses

This study was guided by the Reach, Effectiveness, Adoption, Implementation and Maintenance (RE-AIM) Framework [12], and semi-structured interviewed guides were devised accordingly. Guides were further developed based on interactions between researchers and YMCA-USA constituents (national governing body of LIVESTRONG at the Y) to solicit their feedback and include them in the interview design. We also sought input from program directors, instructors and survivors at one YMCA branch in Worcester, MA on the interview guides. Two guides were developed, pilot-tested (one staff and one survivor) and finalized to solicit information on the recruitment (YMCA staff) and referral (survivor) processes. Telephone interviews were chosen over face-to-face interviews because participants were located across the country. We also chose not to use video conference interviews to lessen participant burden and because we were not soliciting information about participant’s physical reactions to our questions. Participants expressing interest in the study were scheduled for a telephone screening, and upon successful completion, all interviewees were read the informed consent, and provided their verbal consent to participate in the interview. Interview guide questions for both groups, program staff and survivors, are shown in Table 1. Probes and follow-up questions were used as needed to allow participants’ to elaborate or ask any questions they had. All participants who completed the interview received a $25 gift card for compensation.

**Table 1:** LIVESTRONG at the Y staff and survivor interview guide questions and prompts

**LIVESTRONG at the Y Staff Questions**	
1. How does the recruitment process for getting new participants in the program work on your end?	
a. How does it work on the participant’s end?	
b. What approaches have you found most successful?	
c. What has been the biggest challenges for you?	
2. How do most of your participants hear about the program?	
3. If you could design the ideal recruitment/referral process to get new survivors to the program what would it be?	
*Prompt:* When, how and from who would you want survivors to hear about the program? In-person, paper, electronic referrals?	
4. Is there anything else you think is important for us to know to better improve the program’s awareness? Including to providers and survivors?	
**Survivor Questions**	
1. How did you hear about LIVESTRONG at the Y?	
2. Are there any other ways you heard about the program?	
3. Did anyone on your medical team discuss the program with you?	
a. (If yes) What types of things did they discuss with you?	
b. (If yes) When did they discuss this in your treatment?	
4. What types of things would have made it easier for you to learn more about the program?	
5. How did the enrollment process work for you (initial contact, follow-up etc.)	
6. If you had a close family member interested in the program, what would you tell them to do?	
7. If you could design the ideal process to refer your family member or others to the program what would it be?	
*Prompt:* When and how would you want your family to hear about the program?	

Other prompts that were used: “Tell me more”, “How”, “Why”, “And Then”, “Describe that”, “Can you give me an example of that”, “How did you feel about that”, “Why is that important to you”

All interviews were digitally recorded and transcribed verbatim, with any personal information deleted to preserve anonymity*.* We used thematic analysis to identify, code, analyze and report themes within the data [13]. Data were transcribed verbatim and checked all transcripts for accuracy. Two study team members then began initial generation of codes (JF and DM) and used MAXQDA (version 10) to organize content and code transcript data. Transcripts were coded separately for LIVESTRONG at the Y staff and survivors. We performed preliminary coding as interviews were completed, assessing for saturation. We made comparisons between study team members to reconcile differences between codes using the constant comparison method [14]. Transcripts were re-visited to see if new codes identified from more recent transcripts applied to prior coded interviews. Discrepancies in coding were discussed initially between the two coders followed by the larger team and reconciled. Themes derived from the data were generated from codes focusing on experiences, perceptions, facilitators, and barriers to program referrals. Illustrative quotes for each theme are highlighted in the text below. Descriptive statistics were used to describe the demographic characteristics of the participants and facilities.

## Results

Overall, 28 people participated in the interviews including 8 former program participants and 20 program staff. Of the program staff, 16 were program directors responsible for overseeing the programs recruitment and enrollment. The 4 program instructors were primarily involved in session programming and session planning, though also contributed to the recruitment and enrollment efforts. Program staff were from 8 states (Connecticut, Indiana, Maine, Massachusetts, New York, Oregon, Texas, and Wisconsin). Eighty percent (*n* = 16) were female and had worked with the YMCA for a mean of 4.75 (2.34) years (see Table 2). Survivors resided in 3 states (Indiana, Massachusetts, and Wisconsin), were majority female (62.5%) and had completed the program a mean of 1.44 (SD 0.77) years prior. Fifty percent (*n* = 4) of survivors heard about LIVESTRONG at the Y from their cancer care provider, 25% (*n* = 2) from family/friends and 25% (*n* = 2) from a cancer support group.

**Table 2:** Characteristics of current LIVESTRONG at the Y staff and survivors who previously participated in the program

Variable	N (%) or Mean (SD)
**LIVESTRONG Staff (*n*** **= 20)**	
Role	
Program director	16 (80%)
Instructor	4 (20%)
Gender	
Male	4 (20%)
Female	16 (80%)
Years working with LIVESTRONG	4.75 (2.34)
State	
Massachusetts	9 (45%)
Other states	10 (55%)
**Survivors (*n*** **= 8)**	
Gender	
Male	3 (37.5%)
Female	5 (62.5%)
Years since completing LIVESTRONG at the Y	1.44 (0.77)
State	
Massachusetts	6 (75%)
Other states	2 (25%)
How they heard about LIVESTRONG at the Y	
Family/friend	2 (25%)
Support group	2 (25%)
Cancer provider	4 (50%)

For program staff interviews, 4 themes emerged: 1) Program awareness should be further developed for both the general public and medical providers; 2) Strong relationships with medical providers increased program referrals; 3) Electronic referral systems between providers and LIVESTRONG would help to streamline the referral process; and 4) Bi-directional communication between program staff and medical providers is key to providing patient progress updates. Sample illustrative quotes are presented with each theme below:

### Program awareness should be further developed for both the general public and medical providers

Most program directors and instructors reported the need for more general public awareness about the LIVESTRONG at the Y, especially for those who are not members of a YMCA. One program director noted:

*“The biggest thing, too, that’s lacking is really our promotion of the program. So, I know it seems like – of course people know about LIVESTRONG, but I’m here, and I see it, and I’m part of the Y. But anyone I ask outside of here from one of the different state – my friend moved to Texas. Her husband got cancer. She had no idea what LIVESTRONG was. So is it just ‘cause they don’t go to the Y, they don’t know about the Y?”*

*An instructor concurred:*

*“I think a lot of it is just building that awareness that the program exists, because there are plenty of people that could take advantage of it in our area within the seven counties that we serve, but the word really isn’t out there, and I think that’s what I need to do.”*

### Strong relationships with medical providers increased survivor referrals

Many program directors and instructors reported that they had relationships with some medical providers in their area to help facilitate referrals. These included a number of providers, including oncology, primary care, and nurse navigators. One staff member noted:

*“So we send them (oncologists, primary care) the information, and then a lot of it is people that are still in treatment go back and talk to their oncologists how great this program has been for them, and then the oncologist sees that and really starts speaking toward it. So we have a lot of support in that sense as well.”*

Another program director agreed:

*“We work with some nurse navigators, and actually it’s been kinda nice, because the nurse navigator that sends us the most referrals has went through our program as well. So she’s a huge advocate for our program. We have an oncologist in the area that is a huge advocate for our program as well and tells all of her patients about it. And then a cancer service center in the area puts our information and our class list, upcoming class list, in their newsletter every month. So, that’s been helpful as well.”*

A few program directors spoke to their efforts to educate providers, including educational sessions and visiting sites/sessions to view what participants do in the program.

*“it’s word of mouth, doctors, the ones that we do have that connection with our program. We have a signature event in November at our hospital – and those’re a lotta the oncology doctors. So they’ll do referrals, but it’s not all doctors definitely know about it.”*

Another program director concurred:

*“I’ve talked to nurse navigators, and I asked, “Why don’t you push or program?” Come and see what it is I want you to push for your patients. If you wanna go through the entire intake process, I will take you through that so that you know what they can expect. If you wanna see a class who’s running, come and play with us. Bring some tennis shoes. Come work out with us. Come watch us from the doorway, whatever it is you wanna do, talk to our participants, anything.”*

### Electronic referral systems between providers and LIVESTRONG at the Y would help to streamline the referral process

Several instructors and program directors reported that using an electronic referral system would save time and provide ease for providers to refer patients to programs:

*“If we could do doctor referrals online... There’s just a link for them to go do all this, that’s – saves a lotta time on our end.”*

*“I know we’ve even looked into a system which connects doctors and patients to different programs, depending on their condition, what outcome they’re looking for and their location. So, we haven’t really progressed as far as how we can apply it. But the intention is to find some way using technology to make this process easier and really value the time that we have.”*

*“I think maybe the patient portal would be awesome or, a mass e-mail from the hospital –I’ve noticed is if it comes from their medical staff … they are more likely to try it, to sign up for it.”*

One program director noted the importance of being able to include personalized feedback on the patient’s status into the referral form.

*“Having an electronic referral system, where I could get a little popup window that somebody’s been referred through a secure network, here’s a medical release and the contact information. If I get medical releases without any sort of information about a patient … I really value that intake to see if this is the right fit for them at that time.”*

One program director reported hearing of others using an electronic referral system for providers to directly refer patients to the program:

*“Some of them (doctor’s) are starting to send an actual medical referral over to us saying that they told somebody that they need to be in this program. … I know some other Y’s actually did that in an electronic referral system. Their doctors or their cancer center made them a referral option, and they send referrals that way to them.”*

### Bi-directional feedback between program staff and medical providers is key to providing patient progress updates

Program directors reported wanting to provide feedback to providers in real-time, using an electronic platform in an ideal referral scenario:

“*… thinking about some kind of platform, too, that really allowed a provider to see what their patient is doing, kinda almost in real time, some kind of electronic system so that it doesn’t have to be this fax and call and things of that nature...”*

Some program directors reported having success using a system to provide feedback to their participants’ providers:

*“… we also give them (oncologists) a feedback report as well. We created a generalized feedback report, which says, “Your patient, date of birth, enrolled or completed in X program on this date,” or, “Program was unable to participate. Participant dropped out,” … so that the oncologist or the physician or whoever knows where the patient ended up and have that feedback report from us”.*

Another program director suggested increased communication led to greater referrals:

*“… one of the things that we incorporated that was really beneficial … when our referrals started to take off is we created a report pre- and post-assessment of the fitness-assessment data. And we faxed it to the doctors with the participants’ permission. That’s when we really started to see doctors referring.”*

For survivors interviews, 3 themes emerged: 1) Survivors trust their medical team and the information they provide about physical activity; 2) Providers need to incorporate an action plan and referrals for survivors to be active once treatments are completed; and 3) Personal experiences of those who participated in LIVESTRONG at the Y resonate with survivors and increase participation. Sample illustrative quotes are presented with each theme below:

### Survivors trust their medical team and the information they provide about physical activity

Half of the survivors interviewed heard about the program through a medical provider. Many credited their medical team and providers for decisions made throughout their treatment. They emphasized how important their providers were and the magnitude of the information they would relay to them:

*“Well, certainly hearing from it from my medical team that everybody – I assume everybody pays close attention to their medical team, especially when you get a diagnosis of cancer. It’s serious. So you listen to ‘em. You pay attention to them.”*

Another survivor noted providers are best-suited to make referrals:

*“I believe that people, docs, nurses, PCAs in the clinic should be made aware of it, maybe even a little seminar or something, “Hey, this is available to your patients. Please, when you’re speaking to them, mention it to them. Give ‘em a brochure.” It’s a matter of getting the word to people. Some of your people in cancer treatments got enough on their mind to think about some other thing, but if somebody presented to them, then certainly that’s gonna help”.*

Survivors who didn’t hear about it from their medical provider felt that it would’ve been helpful to get a referral from them:

*“It might’ve been helpful to have been handed something from either the doctor, the oncologist, or the hospital shrink they made me go talk to. – I spent a lotta time online.. a lot of the cancer links bring you to the LIVESTRONG organization. And nowhere did I see something (about LIVESTRONG at the Y) … ..It must be overwhelming to people to be told, “We’ve gotten rid of the cancer, but it might come back, but now you need to get yourself in shape,” and not really understand what that all involves, the life changes that that all involves.”*

### Providers need to incorporate an action plan and referrals for survivors to be active once treatments are completed

Most survivors reported wanting to hear about programs available to them when they completed their treatment. They mentioned the timing of after completing treatment or after a transplant as being the opportune time for medical providers to intervene.

*“I can only speak for myself from a transplant standpoint … the doctor, the PCA, the nurse, somebody from pharmacy, they come into your room to get you ready to leave and go home. Well, it’s part of that whole discharge process. Shouldn’t that be in there about LIVESTRONG?”*

Another survivor concurred about their last appointment at their clinic:

*“… it was an exit appointment almost, not quite an exit interview, but I think about it similarly like a survivor, “Here’s your next steps after you’ve gone through surgery …*. T*his is offered to you.” So at (name) clinic it was a nurse, and then it was – at my local cancer center – I believe she’s also a nurse, but it was a survivorship appointment, where we reviewed my diagnosis and the treatment that I had, and moving forward, what’re my next steps, and then also here’s a program that’s offered for you.”*

One participant noted that she would like it to come from her primary care doctor, as her final visit with her oncologist was not helpful.

*“I think probably primary care would be the best and that’s a person that you probably pretty much would trust or at least give credence to it and know if the next time you see them they’re gonna ask you, “Did you do that? Did you think about there’s gonna be a follow-up?” whereas, that oncologist, they’re not gonna follow up on that.(Name) clinic goes through this. It’s sort of a perfunctory final visit, and I have to tell you – it was so unimpressive. I can’t even tell you what they covered, but it was this long interview. I don’t know what was the purpose. I think it was for collecting data, not for really helping people … and it was boring and useless.”*

They also wanted to know from their provider if they were physical ready and able to complete a program like LIVESTRONG, not knowing exactly what it entailed.

*“I would think that if there was some sort of contact made through your medical group … When you’re at the point when you begin to say, “How am I gonna recover from this? How am I gonna get back to normal physically?” is when it would be helpful to have someone have a routine call to say, “We have this available.” … I guess that you’d have to do it through your doc, because how would the Y know that you were a candidate?”*

Another survivor concurred about how it would’ve been easier to be referred to LIVESTONG:

*“The thing that would’ve made it easier was what we would do and what the program was – how it was structured and if I was physically ready for it. I think that was my big unknown. And then after the first meeting or session, I guess, all of my fears were put completely at ease. It was like, we’re gonna help you do what you can do in this way. So that was really helpful, but I was definitely curious before we started. What are we getting into here, and will I be able to do it?”*

### Personal experiences of those who participated in LIVESTRONG resonate with survivors and increase participation

Several participants reported being able to refer other people they knew who had cancer to the program would be helpful as it was to them.

*“they (LIVESTRONG) always send out at the beginning of the year, “Hey, here’s our schedule. If you know anybody with cancer” – and I have referred some people to the program.”*

Some survivors noted that hearing personal experiences from other survivors would be helpful in understanding what the program consisted of and how it could relate to them.

*“I would say that if you have all the materials and all of those things, with all the technology right now, I can forward that information to that person, because I’ve been there. Even if all those information are there, I think there is such a thing about speaking personally to that person, telling your experience, and it makes a lotta difference, right?.”*

Survivors notes the description of the program would be helpful when being referred:

*“… but it’s the specific nature of LIVESTRONG. If that is described to people, I think it has a much greater chance of success than just in general describing it as a “get back and get fit” program. The fact that it covers every single possible way that a body could move and get back and fit and that you get to try it all, that’s the Number 1 thing about that program that makes it really the success that it is or the success for me that it was.”*

## Discussion

This study was the first to examine perceptions and experiences of recruitment and referrals to a community-based physical activity program for cancer survivors from staff and participant perspectives. Twenty LIVESTRONG at the Y staff and 8 survivors completing the program participated in semi-structured interviews exploring their experiences with program referrals. Both staff and survivors described low levels of awareness of LIVESTRONG at the Y across multiple levels; knowledge and awareness were perceived as low among the oncology teams, the broader medical care teams and the greater lay community. Many staff reported rigorous outreach to providers through in-services, emails, flyers, phone calls and session visits. However, both staff and survivors noted the preferred method of referrals from and communication with providers was through an electronic referral system. Survivors also made note of the importance of identifying with other survivors in order to increase participation in the program.

Program directors discussed many ways in which they attempted outreach and education to providers, though felt it was difficult to communicate with them. Consistent with our findings, prior studies showed a lack of effective ways to communicate physical activity education and awareness to cancer care providers [15, 16]. However, evidence also suggests providers are receptive to educational sessions and/or having exercise specialists on the clinical team, thinking they would be helpful [6]. Staff also noted the need for a standardized referral system, preferably electronic, while being able to have bi-directional communication with providers. As health reforms have emphasized physician use of electronic medical health records for surveillance and referral patterns [17] and patients become more adept at using technology, one solution is to integrate a referral system into a technology-based referral platform or patient portal (electronic medical records). One study examining provider facilitators of survivors to community-based physical activity programs found that 96% of providers wanted a standardized referral process and form, 90% wanted an online/electronic referral process, and 75% wanted confirmation their referral was received [10]. Other studies showed that providing physical activity information plus referrals to patients was more effective in increasing physical activity program participation and improved health outcomes as compared to information alone [18–20]. One community-based physical activity program offered in Canada and facilitated by YMCA receives referrals directly from oncologists [21]. However, this program is only operated at one YMCA site unlike LIVESTRONG at the Y which is offered at over 800 YMCA sites. Further investigation is warranted to work with both program staff and clinical providers to ensure the design of appropriate referral pathways into both party’s workflows. This may potentially address aforementioned provider referral barriers and facilitators, while easing the burden of outreach on community workers who may already be spread thin.

Most survivors preferred to hear from someone on their oncology team, though one survivor preferred to hear from her primary care physician due to the continued follow-up care she would receive from them. Many of LIVESTRONG at the Y staff reported strong relationships with oncologists and those in the oncology clinic as compared to primary care physicians. Transition of care between oncology and primary care has been a longstanding gap in care [7]. Cancer survivorship plans and transition to primary care varies widely and have not been guided by a standardized process [22]. The National Comprehensive Cancer Network (NCCN) has suggested surveillance and referral patterns should be standardized, begin during cancer care and be carried into primary care [23]. The NCCN has proposed a standard Survivorship Screening Questionnaire be integrated into survivorship care annually by oncology or primary care providers, including physical activity assessments [23]. Integrating these assessments into electronic medical records will allow for transferability between providers and continued long-term follow-up care. Integrating electronic referrals and communication between program staff and the current provider may also address survivor preferences of receiving electronic referrals and program staff preferences of bidirectional feedback with providers in real-time.

Though we found medical providers to be the top and preferred referral source, many participants (and program staff) mentioned the importance of relating to those who had completed the program. Peer support may provide emotional, informational and appraisal support to others and has been shown to be beneficial during weight management, alcohol and smoking cessation programs [24, 25]. Specific to survivors, peer support has shown to be effective at increasing participation in other community-based physical activity programs, though the majority have been implemented by researchers and need to further investigate sustainable dissemination in the community [26]. Survivors also noted the importance of companionship, motivation and health promotion from their peers [27]. Being able to receive a referral from a medical provider coupled with information from a LIVESTRONG at the Y champion or advocate may be one way to effectively increase survivor enrollment in LIVESTRONG at the Y. Prior work with cancer survivors and technology-assisted physical activity interventions has shown survivors preferred support from family, peer coaches [28] and health care providers [29, 30].

There are several limitations to this study. Snowball sampling for instructors and program graduates may have led to unbalanced demographic characteristics of the population. However, since we only used snowball sampling to reach instructors and survivors participating in the program, we were still able to reach a more diverse population of YMCA Associations overall. Due to this approach, we also do not have access to the number of emails sent out to potential instructors and survivors and are unable to calculate the response rate for these two groups. This approach may also lead to selection bias. It is possible that those who had good/better experiences with the program (staff and participants) were more likely to respond, thus limiting participant representativeness. As previously noted, more than half of our participants were from Massachusetts and responses may differ between states based on state guidelines, steering committees, and action plans. We also only interviewed a small portion of survivors who took part in the program. Which may limit our generalizability. To generalize our data, we used a representative sample of both high and low household YMCA areas, though future trials should assess variations in participant responses by household income status. Future research will seek information from those who were made aware of the program but did not participate, or those who were never made aware of the program. We interviewed a large sample of program staff, though concluded our instructor interviews after six to prioritize reaching program directors, as they had more control over the recruitment/referral process.

## Conclusion

As the number of cancer survivors continues to grow, efforts must be made to address reported patient, clinical and community-based program barriers to increasing survivor’s physical activity. A systematic surveillance, education, referral and follow-up pattern integrated into clinical workflow of oncology, in coordination with primary care, may increase the number of survivors referred to evidence-based programs. This may be augmented by the support of peer cancer survivor champions or advocates during the referral process. As stated by Courneya and colleagues, it is critical to design approaches to translate physical activity in cancer survivorship into “real world settings” using dissemination and implementation research [15]. These approaches should include but are not limited to: (a) optimal strategies for obtaining stakeholder buy-in within the clinical organizations, (b) effects of integrating PA counseling and referral into the survivorship care plan and/or the electronic health record (e.g. impact on prompting and changing clinical practice), and (c) implementation approaches for integrating exercise professionals into the health care setting and referral pathways. This study provides important insight into referral preferences of cancer survivors and staff to a community based physical activity program for cancer survivors as well as facilitators to implementation. Future work should include understanding barriers to clinical workflow and use of technology as a referral source for patients and providers.

## Data Availability

The datasets during and/or analyzed during the current study available from the corresponding author on reasonable request.

## Abbreviations

RE-AIM: Reach effectiveness adoption implementation maintenance
